# Supplementary material for: The Construction and Exploration of a Comprehensive MicroRNA Centered Regulatory Network in Foxtail Millet (Setaria italica L.)
Source: Front Plant Sci. 2022 May 6;13:848474. doi: 10.3389/fpls.2022.848474 (PMC9121102; doi:10.3389/fpls.2022.848474)
Supplement: Supplementary file 3 [file Data_Sheet_3.zip › Sit-miRN3027_Seita.9G416900.1_3120_TPlot.pdf]

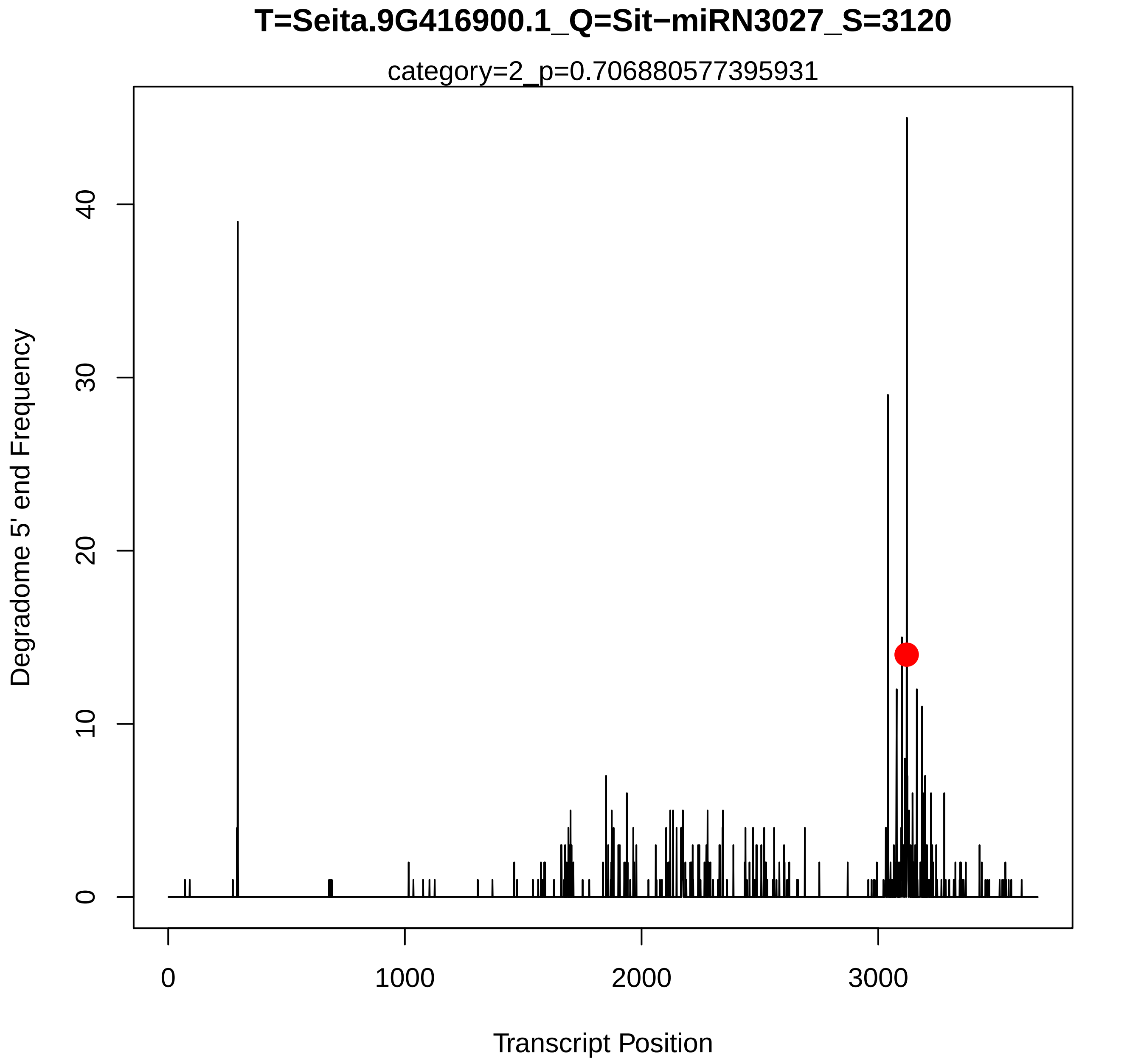

T=Seita.9G416900.1_Q=Sit−miRN3027_S=3120
category=2_p=0.706880577395931
Degradome 5' end Frequency
0
10
20
30
40
0
1000
2000
3000
Transcript Position
